# A Comparative Study of Human and Zebrafish Pregnane X Receptor Activities of Pesticides and Steroids Using *In Vitro* Reporter Gene Assays

**DOI:** 10.3389/fendo.2021.665521

**Published:** 2021-05-18

**Authors:** Nicolas Creusot, Clémentine Garoche, Marina Grimaldi, Abdelhay Boulahtouf, Barbara Chiavarina, William Bourguet, Patrick Balaguer

**Affiliations:** ^1^ Institut de Recherche en Cancérologie de Montpellier (IRCM), Inserm U1194, Institut Régional du Cancer de Montpellier (ICM), Université Montpellier, Montpellier, France; ^2^ Centre de Biologie Structurale (CBS), Inserm, CNRS, Université Montpellier, Montpellier, France

**Keywords:** pesticides, steroids, reporter cell lines, human PXR, zebrafish PXR

## Abstract

The nuclear receptor pregnane X receptor (PXR) is a ligand-dependent transcription factor that regulates genes involved in xenobiotic metabolism in mammals. Many studies suggest that PXR may play a similar role in fish. The interaction of human PXR (hPXR) with a variety of structurally diverse endogenous and exogenous chemicals is well described. In contrast, little is known about the zebrafish PXR (zfPXR). In order to compare the effects of these chemicals on the PXR of these two species, we established reporter cell lines expressing either hPXR or zfPXR. Using these cellular models, we tested the hPXR and zfPXR activity of various steroids and pesticides. We provide evidence that steroids were generally stronger activators of zfPXR while pesticides were more potent on hPXR. In addition, some chemicals (econazole nitrate, mifepristone, cypermethrin) showed an antagonist effect on zfPXR, whereas no antagonist chemical has been identified for hPXR. These results confirm significant differences in the ability of chemicals to modulate zfPXR in comparison to hPXR and point out that zfPXR assays should be used instead of hPXR assays for evaluating the potential risks of chemicals on aquatic species.

## Introduction

In the context of increasing exposure to environmental contaminants, the detoxification process plays a critical role in the protection of human and wildlife. To this end, the pregnane X receptor (PXR), as a nuclear receptor, is a key transcriptional factor that regulates the expression of a wide range of genes coding for metabolic enzymes (*e.g.* P450, GST, UGT) and transporters (*e.g.* MDR1, MRP, OATP2) involved in the elimination of adverse chemicals and the clearance of endogenous hormones to sustain homeostasis in mammalian species (1). Several studies also highlighted its role in bone homeostasis, inflammation, drug-drug interaction, cancer drug resistance and proliferation in humans (2). In fish, although PXR role remains unclear, recent investigations reported its involvement in the regulation of P450 genes and revealed a crosstalk between zebrafish Aryl Hydrocarbon Receptor (AhR) and PXR, suggesting a role in the detoxification of xenobiotics in addition to bile salt metabolism (3–6). Thus, modulation of fish PXR could affect their ability to eliminate adverse chemicals. In this context, it is crucial to identify environmental modulators of PXR to unravel the environmental hazard/risk implications for fish.

PXR has been cloned and functionally characterized in various species such as human, rat, polar bear, zebrafish, and frog (7). These investigations revealed marked interspecies differences in terms of ligand selectivity and specificity in parallel with high cross-species sequence divergence in the ligand binding domain (7, 8). The ligand binding pocket (LBP) of the human PXR is smooth, hydrophobic and large (~1300 Å) (9) allowing to be promiscuously activated by endogenous hormones and a broad number of chemically and structurally different compounds including environmental contaminants, in accordance with its role in the xenobiotic detoxification. These compounds include steroids, pesticides, alkylphenols, bisphenols plasticizers, pharmaceuticals, etc. (10–14). Besides, we have recently described the capacity of environmental pollutants to bind simultaneously to the hPXR, leading to its synergic activation (15, 16). Since X-ray crystallography of the PXR has only been reported for human, little is known regarding the structure of the PXR in other species. Nevertheless, homology models of the ligand binding domains (LBDs) of zebrafish (~1000 Å) predicted smaller LBPs than that of hPXR (8). Despite little evidence, the fish PXR seems to be activated by less chemicals, likely because of its smaller binding pocket (7, 8, 17). In addition, homology models combined to docking studies showed that amino-acid residues involved in ligand interaction differ between species (8, 17). Altogether, these results showed that functional analysis of zebrafish PXR would be particularly useful in defining ligand specificity in terms of efficacy and potency in fish.

The aim of this study is to better characterize the cross-species differences between human and zebrafish in the modulation and the function of the PXR. To this end, we first screened a set of chemically and structurally various compounds (steroids and pesticides) using human stable reporter gene cell lines based on chimeric (Gal4-PXR) receptors. We then confirmed the zfPXR potency in a zebrafish reporter cell line. Finally, we investigated the structural basis of the differences in PXR modulation between human and fish applying homology docking models.

## Material and Methods

### Chemicals and Reagents

A list of tested chemicals is provided in the **Supplementary Material** (**Supplementary Table 1**).

### Plasmids

The total zfPXR coding sequence (M1-T430) was isolated from the ZFL cells extracts by RT-PCR with primers containing XhoI and KpnI restriction enzyme sites (**Supplementary Table 2**). The full-length sequence of the zfPXR was then integrated in the pSG5-puromycin plasmid between XhoI and KpnI sites. Primers were then designed to amplify the ligand binding domain (LBD) of the zfPXR (M111-T430). The LBD sequence of the zfPXR was then integrated in the pSG5-Gal4(DNA-binding domain (DBD))-puromycin plasmid between XhoI and SacI enzyme restriction sites.

The PGL4.24-6xPXRE reporter plasmid (6) is a kind gift from Anke Lange and Charles Tyler. The PXRE6-TATA-luciferase-hygromycin was constructed by adding the hygromycin resistance in this plasmid.

### Establishment of Stable PXR-Based Reporter Gene Cell Lines

The cell lines used in this study are listed in **Supplementary Table 3**. The HG5LN, HG5LN Gal4-hPXR and HG5LN Gal4-zfPXR cell lines were previously described (19). Briefly, HeLa cells stably transfected with the GAL4RE_5_-βGlobin-Luc-SVNeo plasmid (HG5LN cell line) (15). HG5LN cells were stably transfected with the pSG5-GAL4(DBD)-hPXR(LBD)-puro or pSG5-GAL4(DBD)-zfPXR(LBD)-puro plasmids. HG5LN Gal4-hPXR and Gal4-zfPXR cell lines were selected for their inducibility in presence of SR12813 3 μM and clotrimazole 1 μM, respectively.

The ZFL zfPXR cells were obtained by stable cotransfection of ZFL cells with the pSG5-zfPXR-puromycin and PXRE6-TATA-luciferase-hygromycin plasmids and selected for their inducibility in presence of clotrimazole 1 μM.

### Cell Culture

HG5LN cells were cultured in Dulbecco’s Modified Eagle Medium: Nutrient Mixture F-12 (D-MEM/F-12) containing phenol red and 1 g/L glucose and supplemented with 5% fetal bovine serum, 100 units/mL of penicillin and 100 µg/mL of streptomycin supplemented with 1 mg/mL geneticin in a 5% CO_2_ humidified atmosphere at 37°C. HG5LN GAL4-hPXR and GAL4-zfPXR were cultured in the same culture medium supplemented with 0.5 µg/mL puromycin.

ZFL zfPXR cells were cultured in Leibovitz’s L-15, D-MEM, Ham’s F-12 medium (LDF medium) containing 50% L-15, 35% D-MEM, and 15% F-12, 5% FCS with 0.15 g/L sodium bicarbonate, 15 mM HEPES buffer, 0.01 mg/mL insulin, 50 ng/mL EGF, 100 units/mL of penicillin, 100 µg/mL of streptomycin supplemented with 0.5 µg/mL puromycin and 0.25 mg/mL hygromycin B in a humidified atmosphere at 28°C.

### Luciferase Assay

The ability of chemicals to modulate the hPXR and zfPXR was investigated in HG5LN, HG5LN-hPXR, HG5LN-zfPXR, and ZFL-zfPXR cell lines after exposure to serial dilutions of the compounds and measurement of luciferase activity. Exposure of the HG5LN cells which do not express PXR allows to check the specificity of the PXR transactivation in HG5LN-hPXR and HG5LN-zfPXR cell lines. Briefly, cells were seeded in 96-well white opaque flat bottom microplates at 0.5 10^5^ cells per well in culture medium. After 24h of growing at 37°C (HG5LN, HG5LN-hPXR and HG5LN-zfPXR cell lines) or 28°C (ZFL-zfPXR cell line), the culture medium was removed and replaced by DMEM-F12 medium without phenol red (Gibco 21041-025) supplemented with 100 units/mL of penicillin, 100 µg/mL of streptomycin and dextran-coated charcoal-treated fetal calf serum DCC-FCS (5%) (test medium) for HG5LN-hPXR and HG5LN-zfPXR cell lines, and LDF medium supplemented with 100 units/mL of penicillin, 100 µg/mL of streptomycin and dextran-coated charcoal-treated fetal calf serum DCC-FCS (5%) for the ZFL-zfPXR cell line. Cells were then exposed to a concentration range of the compounds by using an automated workstation (Biomek 3000, Beckman Coulter). The final concentration of DMSO in the well never exceeds 0.1% (v/v). After 16h of exposure, medium was removed and 50 µL of test medium containing 0.3 mM D-luciferin were added per well. After 5 min, the production of light was assessed in living cells using microplate reader (MicroBeta, PerkinElmer SAS, Courtabœuf, France).

To assess antagonistic activity, cells were coexposed with serial dilutions of the tested chemicals in presence of 0.3 µM SR12813 (HG5LN-hPXR) or 0.1 µM of clotrimazole (HG5LN-zfPXR and ZFL-zfPXR) that correspond to 80% of the maximal transactivation.

### Data Modelling

In the transactivation assay, each compound was tested at various concentrations in three independent experiments at least. For each experiment, tests were performed in quadruplicates for each concentration, and data are expressed by means values with standard deviations. Dose response curves were established for chemicals at concentrations where they do not modulate luciferase expression in the HG5LN control cell line. Curves were modelled based on the four parameters hill equation using the freely available Excel Macro REGTOX 7.02 (18). This allowed to determine EC50 and IC50 values of tested chemicals.

## Results

### Modulation of the Transcriptional Activity of the zf and hPXR by Various Chemicals

In this study, we evaluated the ability of PXR prototypical ligands, 21 steroids and 80 pesticides (**Supplementary Table 1**), to modulate both zfPXR and hPXR. The chemicals were first tested for non-specific modulation of luciferase expression on the HG5LN parental cell line, which contains the same reporter gene as HG5LN-GAL4-PXRs cells, but lacks Gal4-PXRs (data not shown). Then the chemicals were tested on the HG5LN GAL4-hPXR and GAL4-zfPXR at concentrations that were not able to inhibit or activate luciferase expression in the HG5LN reporter cell line.

**Figure 1:**
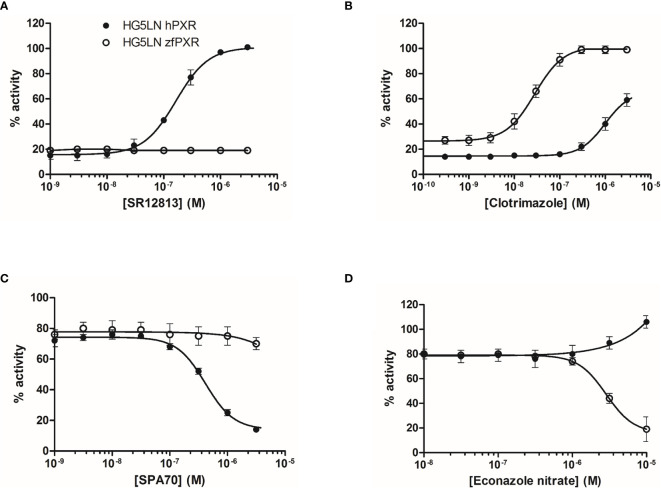
Transcriptional activity of hPXR and zfPXR in response to reference chemicals SR 12813 **(A)**, clotrimazole **(B)**, SPA 70 **(C)**, and econazole nitrate **(D)**. Results are expressed as a percentage of the maximum luciferase activity induced by 3 µM SR 12813 (HG5LN-hPXR) or 1 µM clotrimazole (HG5LN-zfPXR). Error bars represent standard deviations.

The activity of the chemicals on hPXR was compared to the activity of the reference PXR agonist SR12813. This compound fully activated hPXR with an EC50 of 0.16 µM while it was not active in the HG5LN Gal-zfPXR cells (**Figure 1A** and **Table 1**). Rifampicin, another well-characterized hPXR full agonist, was also found exclusively active on hPXR with an EC50 of 0.44 µM (**Table 1**). For zfPXR, the activity of the chemicals was compared to the activity of the reference ligand clotrimazole. This compound activated both the HG5LN Gal-zfPXR and Gal-hPXR cell lines with the half-maximal effective concentration (EC_50_) values of 0.03 µM and 1.0 μM respectively (**Figure 1B** and **Table 1**). SPA70 (20) and econazole nitrate were used as reference antagonists for hPXR and zfPXR, respectively. SPA70 inhibited only hPXR, with an IC50 of 0.41 µM (**Figure 1C** and **Table 1**). Econazole nitrate inhibited luciferase activity in HG5LN Gal-zfPXR whereas it activated it in HG5LN Gal-hPXR cell lines. The EC_50_ for hPXR was 12 μM and the IC50 for zfPXR was 2.8 μM (**Figure 1D** and **Table 1**). Finally, pregnenolone 16α-carbonitrile, a mouse PXR agonist, partially activated hPXR whereas it did not activate zfPXR. Dexamethasone, another mouse PXR agonist, had no effect on hPXR and zfPXR.

**Table 1:** Maximal or minimal activity and EC50 or IC50 of reference compounds on HG5LN-hPXR and HG5LN-zfPXR.

Compound	hPXR agonism		zfPXR agonism		zfPXR antagonism	
	EC50 (µM)	Max (%)	EC50 (µM)	Max (%)	IC50 (µM)	Min (%)
**DMSO**		19 ± 6		28 ± 1.7		
Clotrimazole*	1.0 ± 0.1	59	0.03 ± 0.002	100	n.a.	-
Econazole nitrate	12 ± 3.0	51	n.a.	-	2.8 ± 0.1	16
SR12813	0.16 ± 0.01	100	n.a.	-	n.a.	-
Rifampicin	0.44 ± 0.03	74	n.a.	-	n.a.	-
SPA70*	0.41 ± 0.04	14	n.a.	-	n.a.	-
Pregnenolone16α-carbonitrile	22 ± 9.7	32	n.a.	-	n.a.	-
Dexamethasone	n.a.	-	n.a.	-	n.a.	-

n.a., not active; max, maximal activity of the chemicals obtained at 10 µM except for compounds that were not tested above 3 µM because of toxicity *; min, minimal activity of the chemicals obtained at 10 µM except for compounds that were not tested above 3 µM because of toxicity*. Maximal activities of the chemicals obtained at 10 µM (excepted for clotrimazole which was tested at the maximal concentration of 1 µM) are expressed as a percentage of the maximal induced by 3 µM SR 12813 and 1 µM clotrimazole for HG5LN-hPXR and HG5LN-zfPXR, respectively. Antagonism assays were performed in coexposure with 0.3 µM SR 12813 in HG5LN-hPXR cells and 0.03 µM clotrimazole in HG5LN-zfPXR cells. Minimal activities of the chemicals obtained at 10 µM (excepted for SPA70 which was tested at the maximal concentration of 1 µM) are expressed as a percentage of the maximal induced by 3 µM SR 12813 and 1 µM clotrimazole for HG5LN-hPXR and HG5LN-zfPXR, respectively.

**Figure 2:**
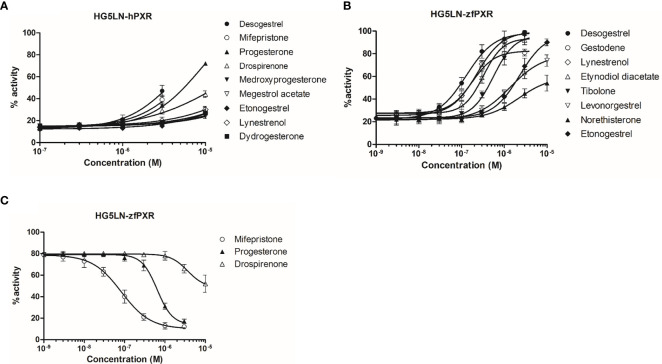
Transcriptional activity of hPXR and zfPXR in response to steroids. **(A)** Agonist steroids on HG5LN-hPXR; **(B)** Agonist steroids on HG5LN-zfPXR; **(C)** Antagonist steroids on HG5LN-zfPXR. Results are expressed as a percentage of the maximum luciferase activity induced by 3 µM SR 12813 (HG5LN-hPXR) or 1 µM clotrimazole (HG5LN-zfPXR). Error bars represent standard deviations.

We evaluated the ability of 21 progestins to modulate both hPXR and zfPXR (**Table 2**). Among them, 9 modulated the activity of hPXR whereas 11 modulated that of zfPXR. All the active steroids on hPXR were agonists with EC_50_ values comprised between 4.2 μM (desogestrel) and 41 µM (lynestrenol) (**Figure 2A** and **Table 2**). On zfPXR, 8 steroids were agonists of the zfPXR with EC_50_ values between 0.13 µM (desogestrel) and 2.3 µM (etonogestrel) (**Figure 2B** and **Table 2**). Surprisingly, 3 steroids were antagonists of the zfPXR with IC_50_ values comprised between 0.08 µM (mifepristone) and 3.6 µM (drospirenone) (**Figure 2C** and **Table 2**). These results indicate that progestins have a preferential selectivity for zfPXR rather than hPXR and that zfPXR is more prone to be antagonized than hPXR.

**Table 2:** Maximal or minimal activity and EC50 or IC50 of progestins on HG5LN-hPXR and HG5LN-zfPXR.

Compound	hPXR agonism		zfPXR agonism		zfPXR antagonism	
	EC50 (µM)	Max (%)	EC50 (µM)	Max (%)	IC50 (µM)	Min (%)
**DMSO**		19 ± 6		28 ± 1.7		
Chlormadinone acetate	n.a.	-	n.a.	-	n.a.	-
Desogestrel*	4.2 ± 0.2	54	0.13 ± 0.01	99	n.a.	-
17α,20β-Dihydroxy-4-pregnen-3-one	n.a.	-	n.a.	-	n.a.	-
Drospirenone	18 ± 1.4	44	n.a.	-	3.6 ± 0.4	52
Dydrogesterone	n.m.	27	n.a.	-	n.a.	-
Ethisterone	n.a.	-	n.a.	-	n.a.	-
Etynodiol diacetate*	n.a.	-	0.32 ± 0.02	92	n.a.	-
Etonogestrel	24 ± 4.6	27	2.3 ± 0.2	90	n.a.	-
Gestodene*	n.a.	-	0.18 ± 0.01	81	n.a.	-
Gestonorone	n.a.	-	n.a.	-	n.a.	-
Levonorgestrel	n.a.	-	1.4 ± 0.2	74	n.a.	-
Lynestrenol*	41 ± 7.7	30	0.23 ± 0.01	98	n.a.	-
Medroxyprogesterone	18 ± 2.4	50	n.a.	-	n.a.	-
Megestrol acetate	19 ± 4.3	50	n.a.	-	n.a.	-
Mifepristone*	5.9 ± 0.2	39	n.a.	-	0.08 ± 0.003	10
Nestorone	n.a.	-	n.a.	-	n.a.	-
Nomegestrol acetate	n.a.	-	n.a.	-	n.a.	-
Norethisterone	n.a.	-	2.2 ± 0.4	54	n.a.	-
Progesterone	9.1 ± 2.2	72	n.a.	-	0.64 ± 0.03	14
Promegestone	n.a.	-	n.a.	-	n.a.	-
Tibolone*	n.a.	-	0.56 ± 0.04	96	n.a.	-

n.a., not active; max, maximal activity of the chemicals obtained at 10 µM except for compounds that were not tested above 3 µM because of toxicity*; min, minimal activity of the chemicals obtained at 10 µM except for compounds that were not tested above 3 µM because of toxicity*. Maximal activities of the chemicals obtained at 10 µM are expressed as a percentage of the maximal induced by 3 µM SR 12813 and 1 µM clotrimazole for HG5LN-hPXR and HG5LN-zfPXR, respectively. Antagonism assays were performed in coexposure with 0.3 µM SR 12813 in HG5LN-hPXR cells and 0.03 µM clotrimazole in HG5LN-zfPXR cells. Minimal activities of the chemicals obtained at 10 µM are expressed as a percentage of the maximal induced by 3 µM SR 12813 and 1 µM clotrimazole for HG5LN-hPXR and HG5LN-zfPXR, respectively.

**Figure 3:**
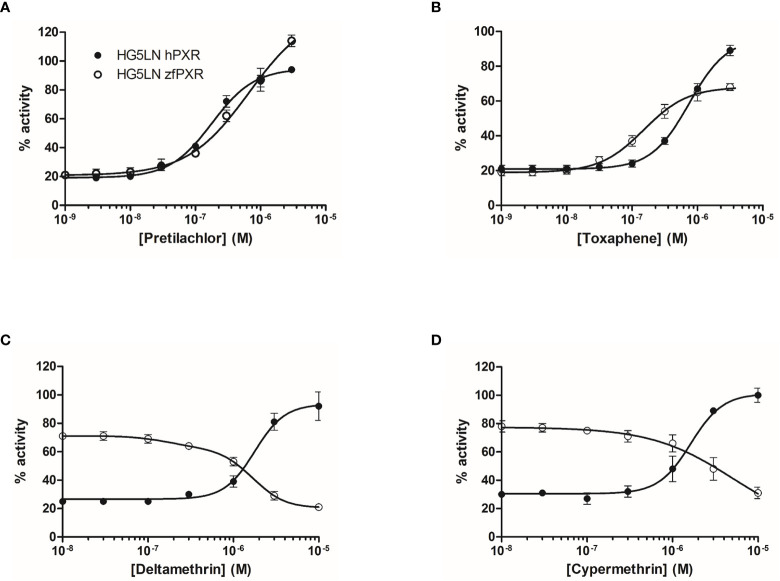
Transcriptional activity of hPXR and zfPXR in response to the pesticides pretilachlor **(A)**, toxaphene **(B)**, deltamethrin **(C)**, and cypermethrin **(D)**. Results are expressed as a percentage of the maximum luciferase activity induced by 3 µM SR 12813 (HG5LN-hPXR) or 1 µM clotrimazole (HG5LN-zfPXR). Error bars represent standard deviations.

We then evaluated the ability of 80 pesticides to modulate both zfPXR and hPXR (**Table 3**). Among them, 61 modulated the activity of hPXR whereas 49 modulated that of zfPXR. All the active pesticides on hPXR were agonists with EC_50_ values comprised between 0.18 µM (pretilachlor) (**Figure 3A** and **Table 2**) and 39 µM (vinclozolin M2) (**Table 2**). On zfPXR, 38 were agonists and 11 were antagonists. The most potent zfPXR agonist was toxaphene (0.15 µM) (**Figure 3B** and **Table 2**) whereas deltamethrin and cypermethrin were the most potent antagonists (**Figures 3C, D** and **Table 2**). These results indicate that pesticides have a preferential selectivity for hPXR rather than zfPXR and that again zfPXR is more easily antagonized than hPXR.

**Table 3:** Maximal or minimal activity and EC50 or IC50 of pesticides on HG5LN-hPXR and HG5LN-zfPXR.

Compound	hPXR agonism		zfPXR agonism		zfPXR antagonism		Compound	hPXR agonism		zfPXR agonism		zfPXR antagonism	
	EC50 (µM)	Max (%)	EC50 (µM)	Max (%)	IC50 (µM)	Min (%)		EC50 (µM)	Max (%)	EC50 (µM)	Max (%)	IC50 (µM)	Min (%)
**DMSO**		19 ± 6		28 ± 1.7			**DMSO**		19 ± 6		28 ± 1.7		
2,4’-DDE	5.5 ± 0.4	78	n.m.	57	n.a.	-	Heptachlor endo-epoxide*	3.4 ± 0.7	69	2.0 ± 0.9	32	n.a.	-
4,4’-DDE	5.6 ± 0.3	77	n.a.	-	n.a.	-	Heptachlor exo-epoxide*	1.9 ± 0.1	85	1.4 ± 0.04	76	n.a.	-
Alachlor	1.5 ± 0.4	76	0.67 ± 0.10	98	n.a.	-	Hexachlorobenzene	n.a.	-	n.a.	-	n.a.	-
Aldicarb	n.a.	-	n.a.	-	n.a.	-	HPTE	n.a.	-	1.8 ± 0.04	37	n.a.	-
Aldrin	7.6 ± 0.3	70	10 ± 4.5	65	n.a.	-	Imazalil*	10 ± 1.4	39	9.8 ± 2.6	43	n.a.	-
Azimsulfuron	n.a.	-	n.a.	-	n.a.	-	Lindane*	10 ± 1.8	62	14 ± 0.9	55	n.a.	-
Bifenox	4.5 ± 0.7	84	5.5 ± 0.4	80	n.a.	-	Linuron	29 ± 3.2	40	n.a.	-	n.a.	-
Boscalid	13 ± 1.1	55	58 ± 82	41	n.a.	-	Mecoprop	n.a.	-	n.a.	-	n.a.	-
Bupirimate*	1.7 ± 0.1	101	22 ± 5.1	26	n.a.	-	Metalaxyl	31 ± 2.6	40	20 ± 3.1	42	n.a.	-
Captan*	n.a.	-	n.a.	-	n.a.	-	Metamitron	n.a.	-	n.a.	-	n.a.	-
Chlordecone*	19 ± 5.6	46	75 ± 23	32	n.a.	-	Methoxychlor*	17 ± 1.7	49	n.a.	-	n.a.	-
Chlorosulfuron	n.a.	-	n.a.	-	n.a.	-	Metolachlor	0.68 ± 0.07	69	0.63 ± 0.03	115	n.a.	-
Chlorpropham*	20 ± 8.6	49	22 ± 16	51	n.a.	-	Mirex	n.a.	-	n.a.	-	n.a.	-
Chlorpyriphos*	2.7 ± 0.4	95	3.0 ± 0.3	101	n.a.	-	Nicosulfuron	n.a.	-	n.a.	-	n.a.	-
Chlortoluron	n.a.	-	n.a.	-	n.a.	-	Oxadiazon	1.1 ± 0.0001	76	n.a.	-	n.a.	-
Cis-chlordane*	8.6 ± 3.0	87	13 ± 0.4	53	n.a.	-	Oxychlordane	4.3 ± 0.3	69	n.a.	-	n.a.	-
Cis-nonachlor*	7.4 ± 3.8	62	n.a.	-	n.a.	-	Oxyfluorfen	3.0 ± 0.1	86	n.a.	-	10 ± 1.2	56
λ-Cyhalothrin	1.7 ± 0.2	107	n.a.	-	6.8 ± 1.0	45	Penconazol	n.m.	42	1.5 ± 0.1	51	n.a.	-
Cypermethrin	1.6 ± 0.2	100	n.a.	-	5.0 ± 0.3	31	Pencycuron*	1.9 ± 0.2	98	n.a.	-	n.a.	-
Cyproconazole	n.m.	44	n.a.	-	n.a.	-	Pendimethalin*	3.0 ± 0.1	91	0.47 ± 0.1	104	n.a.	-
Deltamethrin	1.8 ± 0.2	92	n.a.	-	1.6 ± 0.2	21	Pirimiphos-methyl	22 ± 23	69	1.1 ± 0.1	103	n.a.	-
Diclofop-methyl	n.a.	-	n.a.	-	n.m.	65	Pretilachlor*	0.18 ± 0.02	94	0.58 ± 0.07	123	n.a.	-
Dieldrin*	9.2 ± 1.4	62	n.a.	-	19 ± 8.8	63	Prochloraz*	1.8 ± 0.1	71	n.a.	-	11 ± 5.6	50
Diethofencarb*	19 ± 2.4	45	n.a.	-	n.a.	-	Propiconazole	13 ± 1.5	58	1.0 ± 0.1	33	n.a.	-
Diflubenzuron	n.a.	-	n.a.	-	n.a.	-	Propyzamide	22 ± 1.7	45	n.a.	-	n.a.	-
Diuron	n.a.	-	n.a.	-	n.a.	-	Tebuconazole	6.8 ± 0.4	71	n.a.	-	24 ± 4.3	62
Endosulfan	5.7 ± 0.4	72	11 ± 1.5	57	n.a.	-	Tefluthrin	4.4 ± 0.9	76	4.2 ± 1.1	32	n.a.	-
Endrin*	6.9 ± 1.2	66	14 ± 1.2	57	n.a.	-	Terbutylazine	n.a.	-	n.a.	-	n.a.	-
Epoxiconazole	8.1 ± 0.2	72	1.6 ± 0.1	68	n.a.	-	Thiabendazole*	n.a.	-	n.a.	-	10 ± 0.7	49
Ethoprophos	n.m.	33	n.m.	38	n.a.	-	Thiacloprid	n.a.	-	n.a.	-	n.a.	-
Etofenprox	3.0 ± 0.2	81	n.a.	-	n.a.	-	Thiophanate-methyl	35 ± 7.3	46	n.a.	-	n.a.	-
Fenamiphos	1.7 ± 0.2	76	0.70 ± 0.21	103	n.a.	-	Tolclofos-methyl	11 ± 0.3	59	2.5 ± 0.1	88	n.a.	-
Fenarimol	13 ± 2.2	56	n.a.	-	24 ± 7.1	66	Toxaphene*	0.75 ± 0.03	89	0.15 ± 0.01	68	n.a.	-
Fenbuconazole*	32 ± 13	43	n.m.	43	n.a.	-	Trans-chlordane*	4.3 ± 0.3	92	16 ± 0.8	50	n.a.	-
Fenvalerate*	1.7 ± 0.05	79	n.a.	-	n.m.	66	Trans-nonachlor	5.3 ± 0.2	70	29 ± 5.5	43	n.a.	-
Fipronil*	n.m.	39	n.a.	-	n.a.	-	Triclosan*	11 ± 3.9	62	n.a.	-	n.a.	-
Fipronil sulfone*	5.4 ± 0.4	73	n.a.	-	n.a.	-	Triflurnizole	21 ± 14	51	6.0 ± 0.8	48	n.a.	-
Flufenoxuron*	n.m.	49	8.9 ± 0.6	63	n.a.	-	Vinclozolin	n.a.	-	n.m.	30	n.a.	-
Fluvalinate	1.1 ± 0.1	74	n.a.	-	n.a.	-	Vinclozolin M2	39 ± 9.6	35	n.a.	-	n.a.	-
Heptachlor	n.m.	43	7.4 ± 1.8	44	n.a.	-	Ziram*	n.a.	-	n.a.	-	n.a.	-

n.a., not active; n.m., not modellable; max, maximal activity of the chemicals obtained at 10 µM except for compounds that were not tested above 3 µM because of toxicity or non-specificity*; min, minimal activity of the chemicals obtained at 10 µM except for compounds that were not tested above 3 µM because of toxicity or non-specificity*. Maximal activities of the chemicals obtained at 10 µM are expressed as a percentage of the maximal induced by 3 µM SR 12813 and 1 µM clotrimazole for HG5LN-hPXR and HG5LN-zfPXR, respectively. Antagonism assays were performed in coexposure with 0.3 µM SR 12813 in HG5LN-hPXR cells and 0.03 µM clotrimazole in HG5LN-zfPXR cells. Minimal activities of the chemicals obtained at 10 µM are expressed as a percentage of the maximal induced by 3 µM SR 12813 and 1 µM clotrimazole for HG5LN-hPXR and HG5LN-zfPXR, respectively.

**Figure 4:**
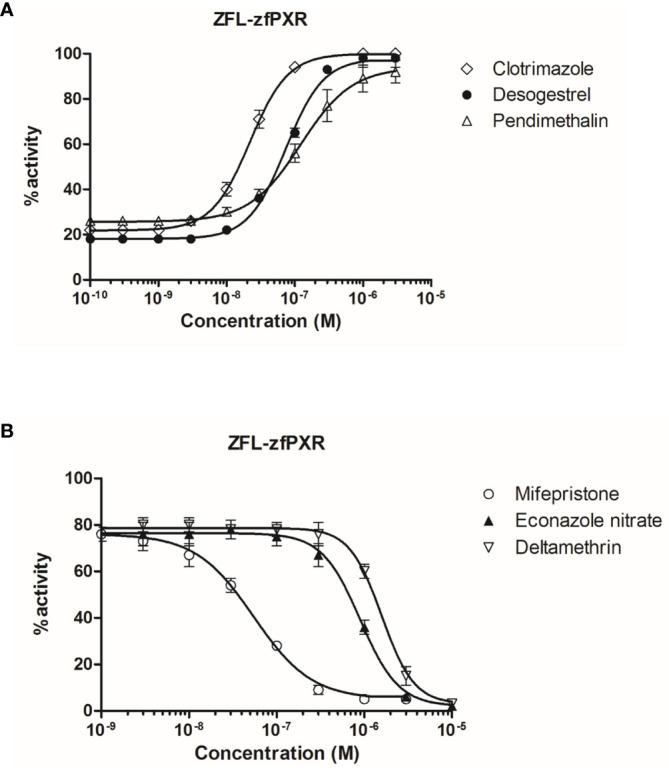
Transcriptional activity of zfPXR in ZFL-zfPXR cells in response to steroids and pesticides. **(A)** Agonist compounds clotrimazole, desogestrel, and pendimethalin; **(B)** Antagonist compounds mifepristone, econazole nitrate and deltamethrin. Results are expressed as a percentage of the maximum luciferase activity induced by 1 µM clotrimazole. Error bars represent standard deviations.

To confirm the zfPXR activity of the chemicals in a zebrafish cellular context, we established a zfPXR zebrafish reporter cell line. We stably cotransfected the zebrafish hepatoma cells ZFL with a pSG5-zfPXR-puromycin and a PXRE6-TATA-luciferase-hygromycine plasmid. In these cells, we tested some of the most active zfPXR agonists and antagonists. EC50s obtained in ZFL-zfPXR cells (0.02, 0.07 and 0.12 µM for clotrimazole, desogestrel and pendimethalin, respectively) were in the same range of those obtained in HG5LN GAL4-zfPXR cells (0.03, 0.13 and 0.47 µM for clotrimazole, desogestrel and pendimethalin, respectively) (**Figure 4A** and **Table 4**). Similarly, IC50s obtained ZFL-zfPXR cells (0.90, 0.06 and 1.6 µM for econazole nitrate, mifepristone and deltamethrin, respectively) are in the same range of those obtained in HG5LN GAL4-zfPXR cells (2.8, 0.08 and 1.6 µM for econazole nitrate, mifepristone and deltamethrin, respectively) (**Figure 4B** and **Table 4**).

**Table 4:** Maximal or minimal activity and EC50 or IC50 of chemicals on ZFL-zfPXR.

Compound	zfPXR agonism		zfPXR antagonism	
	EC50 (µM)	Max (%)	IC50 (µM)	Min (%)
**DMSO**		25 ± 2.3		
Clotrimazole*	0.02 ± 0.002	100	n.a.	-
Desogestrel*	0.07 ± 0.004	98	n.a.	-
Pendimethalin	0.12 ± 0.01	92	n.a.	-
Econazole nitrate	n.a.	-	0.90 ± 0.05	2
Mifepristone*	n.a.	-	0.06 ± 0.004	5
Deltamethrin	n.a.	-	1.6 ± 0.06	3

n.a., not active; max, maximal activity of the chemicals obtained at 10 µM except for compounds that were not tested above 3 µM because of toxicity or non-specificity*; min, minimal activity of the chemicals obtained at 10 µM except for compounds that were not tested above 3 µM because of toxicity or non-specificity*. Maximal activities of the chemicals obtained at 10 µM are expressed as a percentage of the maximal induced by 1 µM clotrimazole. Antagonism assays were performed in coexposure with 0.03 µM clotrimazole. Minimal activities of the chemicals obtained at 10 µM are expressed as a percentage of the maximal induced by 3 µM SR 12813 and 1 µM clotrimazole for HG5LN-hPXR and HG5LN-zfPXR, respectively.

### Structural Analysis of zfPXR Selectivity

**Figure 5:**
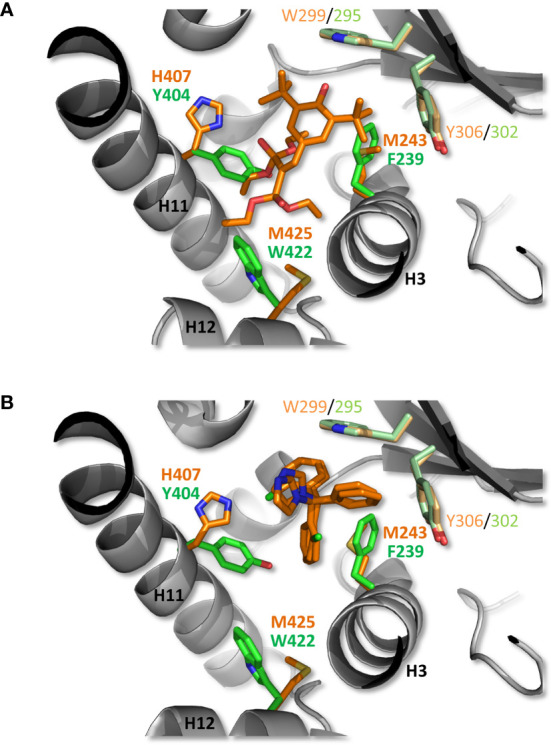
Structural analysis of zfPXR selectivity. A zfPXR LBD model was generated and superimposed on the crystallographic structures of hPXR LBD in complex with the reference agonist SR12813 **(A)** and clotrimazole **(B)**. The ligands and side chains of hPXR residues are shown as orange sticks, while zfPXR residues are colored in green. The conserved π-trap residues in hPXR (W299, Y306) and zfPXR (W295, Y302) are displayed in light orange and light green, respectively. For clarity, only residue differences at key positions are shown.

To gain structural insights into zfPXR ligand binding selectivity, we generated a model of zfPXR LBD using the web-based server EDMon (Endocrine Disruptor Monitoring; http://edmon.cbs.cnrs.fr) (21, 22), and superposed it on the experimental crystal structures of hPXR in complex with SR12813 (23) and clotrimazole (16). As previously observed, the ligand-binding pockets (LBP) of hPXR and zfPXR show significant differences in their size and residue composition (7, 8). In a recent study on the human receptor, we observed that PXR contains an aromatic cage deeply buried at the bottom of the LBP and made up of residues F288, W299 and Y306. This unique aromatic triad (referred to as the π-trap) catches compounds through their most hydrophobic moieties and constitutes the main anchoring point of many compounds, including clotrimazole (16). Interestingly, residues forming the π-trap are fully conserved in zfPXR (**Figure 5**). However, a number of more or less drastic residue substitutions distributed all over the LBPs modulate the interactions between PXR orthologs and chemicals, and account for the differential ligand selectivity of both receptor species. As an example, **Figure 5A** shows how hPXR is able to accommodate SR12813 with strong affinity, while, in contrast, a steric hindrance generated by the replacement of several methionine and histidine residues from helices H3, H11 and H12 by larger and less flexible amino acids prevents binding of SR12813 to the fish receptor. On the contrary, zfPXR is able to accommodate the smaller compound clotrimazole, essentially *via* its interaction with the conserved π-trap. Additional contacts with zfPXR-specific residues such as F239, I280, L281 (not shown) and Y404 may explain the significantly higher affinity of this compound for the fish receptor (**Figure 5B**).

## Discussion

In the present study, we evaluated the ability of 21 progestins and 80 pesticides to alter the transcriptional activity of both zfPXR and hPXR. In accordance with previous studies (8, 24, 25), our data confirmed that clotrimazole is the most potent ligand of the zfPXR (EC_50_ of 0.03 µM), as in other fish species (6, 7). Clotrimazole also activates hPXR but with a lower potency.

Our data also confirmed that the pharmaceuticals SR12813 and rifampicin (hPXR ligands) do not modulate zfPXR as previously reported for carp and zebrafish receptors (6, 8, 10, 25). This observation is in contrast with gene expression studies in carp (4) and zebrafish (26) showing that CYP3A65, a suspected target gene of the fish PXR, is upregulated by rifampicin in these species.

We showed that the modulation of the zfPXR by progestins occurs at lower concentrations than the hPXR modulation. Ekins et al. (8) have previously compared zfPXR and hPXR transactivation by numerous steroids (*i.e.*, estranes, androstanes, pregnanes) and reported a lower promiscuity of the zfPXR for these steroids than the human isoform, whereas they showed quite similar potency and efficacy of these compounds between the two species. Contrary to these previous observations, our study indicates that for progestins, zfPXR is more promiscuous than hPXR. A study including a larger selection of steroids will be necessary to confirm that among steroids, progestins present a zfPXR selectivity. Crystallization of zfPXR in complex with steroids and mutagenesis of the main amino acids in contact with the steroids in the zfPXR pocket would enable to confirm this selectivity. We also showed that the synthetic glucocorticoid dexamethasone and the pregnane pregnenolone 16α-carbonitrile, which are mouse PXR agonists, do not modulate zfPXR in accordance with what was previously reported with carp and zebrafish receptors (6, 8, 27).

Our data also confirmed the low promiscuity of the zfPXR to pesticides compared to hPXR, as previously reported (7, 8, 27). Such discrepancies were also observed in the carp (6) and in the rainbow trout (28). Although less pesticides modulated the zfPXR, we highlighted that the zfPXR was sensitive to some of these contaminants. In particular, clotrimazole was able to modulate zfPXR at low concentrations.

Importantly, out of the 105 chemicals tested (4 reference chemicals, 80 pesticides and 21 progestins), 15 acted as antagonists of the zfPXR while none of them were identified as hPXR antagonists. Moreover, some agonists of the hPXR like mifepristone and econazole nitrate acted as antagonists of the zfPXR. To our knowledge, this study is the first to report the ability of organic chemicals, including environmental contaminants, to negatively regulate the transcriptional activity of a fish PXR. Note in this respect that the first fully validated competitive inhibitors of hPXR have been reported only recently (20, 29). Overall, some of the specific structural features of hPXR (*e.g.* large LBP size and high plasticity) most likely account for its refractoriness to antagonism (16, 30), while in contrast, the smaller and less malleable zfPXR LBP (17) could explain why the zebrafish receptor is more prone to antagonism. Notably, the substitution of a comfortable methionine residue in helix H12 of hPXR for a bulky and rigid tryptophan residue in zfPXR (**Figure 5**) may enable easier destabilization of the active conformation of the activation helix by chemicals. Again, crystallization of zfPXR in complex with RU486 or other zfPXR antagonists would considerably improve our understanding of the mechanism of inhibition of zfPXR.

In human, there is an increasing concern regarding adverse PXR-dependent drug interactions that may be well avoided with suitable PXR antagonists. Recently Chen and coworkers (20, 29) have succeeded to identify a selective and potent hPXR antagonist, SPA70. They failed to crystallize hPXR in complex with SPA70 but succeeded to crystallize hPXR with the chemical SJB7, an agonist closely related to SPA70. The amino acids M425, L428 and F429 from H12 sequence interact with SJB7 and stabilize H12 into an active conformation. Using modelling, Huber et al. (31) proposed that SPA70 fails to stabilize H12 for co-activator binding due to loss of interactions with L428 and F429 (31).

Overall, the promiscuity of PXRs suggests a major role of this receptor in protection of an individual against toxic levels of exogenous (*i.e.*, xenobiotics) and endogenous compounds (hormones, bile salts). From an evolutionary standpoint, the current challenge is to decipher why this receptor has such striking cross-species sequence variation in the LBD and even in ligand-binding residues across mammalian and non-mammalian species (32). This suggests that key ligands of PXR vary across species due to differences either in diet or in physiology. The present study showed that zfPXR is modulated by a variety of ligands but with a lower promiscuity than the hPXR. In particular, it was mainly modulated by progestins. In conclusion, the current study evaluated differences in the activation or inhibition of human and zebrafish PXR by progestins and pesticides and highlighted strong species-specific differences. Furthermore, we observed in a recent study that water extracts could induce different responses in hPXR and zPXR (19) confirming that these receptors are differently activated by environmental chemicals. Altogether, these results indicate that zebrafish nuclear receptor assays should be preferred over human nuclear receptor assays to evaluate the potential risks posed by endocrine-disrupting chemicals to aquatic organisms.

## Data Availability Statement

The original contributions presented in the study are included in the article/**Supplementary Material**. Further inquiries can be directed to the corresponding authors.

## Author Contributions

PB designed research. NC, CG, MG, and WB performed research. AB and BC contributed with new reagents/analytic tools. NC, CG, WB, and PB analyzed data. and NC, CG, WB, and PB wrote the paper. All authors contributed to the article and approved the submitted version.

## Funding

We acknowledge financial support from the French Agency for Food, Environmental and Occupational Health & Safety projects TOXCHEM: N°2018/1/020 (PNR EST) (to PB and WB), the Programme National de Recherche sur les Pertubateurs Endocriniens PestR (PB and WB), and SYNEPEST (PB).

## Conflict of Interest

The authors declare that the research was conducted in the absence of any commercial or financial relationships that could be construed as a potential conflict of interest.

## Abbreviations

PXR, pregnane X receptor; hPXR, human PXR; zfPXR, zebrafish PXR; LBD, ligand binding domain; DBD, DNA binding domain; LBP, ligand binding pocket.
