# Supplementary material for: A Comparative Study of Human and Zebrafish Pregnane X Receptor Activities of Pesticides and Steroids Using In Vitro Reporter Gene Assays
Source: Front Endocrinol (Lausanne). 2021 May 18;12:665521. doi: 10.3389/fendo.2021.665521 (PMC8167039; doi:10.3389/fendo.2021.665521)
Supplement: Supplementary file 1 [file Table_1.docx]

Supplementary Material

**Supplementary Table 1.** Chemical substances used in the study.

| **Chemical** | **CAS** | **Molecular weight (g/mol)** |  | **Chemical** | **CAS** | **Molecular weight (g/mol)** |
| --- | --- | --- | --- | --- | --- | --- |
| **Reference compounds** |  |  |  | **Pesticides** |  |  |
| Clotrimazole ^1^ | 23593-75-1 | 344.8 |  | Endrin ^1^ | 72-20-8 | 380.9 |
| Econazole nitrate ^1^ | 24169-02-6 | 444.7 |  | Epoxiconazole ^1^ | 133855-98-8 | 329.8 |
| SR 12813 ^2^ | 126411-39-0 | 504.5 |  | Ethoprophos ^1^ | 13194-48-4 | 242.3 |
| SPA70 ^3^ | 931314-31-7 | 415.5 |  | Etofenprox ^1^ | 80844-07-1 | 376.5 |
| **Steroids** |  |  |  | Fenamiphos ^1^ | 22224-92-6 | 303.4 |
| Chlormadinone acetate ^1^ | 302-22-7 | 404.9 |  | Fenarimol ^1^ | 60168-88-9 | 331.2 |
| Desogestrel ^1^ | 54024-22-5 | 310.5 |  | Fenbuconazole ^1^ | 114369-43-6 | 336.8 |
| 17α,20β-Dihydroxy-4-pregnen-3-one ^1^ | 1662-06-2 | 332.5 |  | Fenvalerate ^1^ | 51630-58-1 | 419.9 |
| Drospirenone ^1^ | 67392-87-4 | 366.5 |  | Fipronil ^1^ | 120068-37-3 | 437.1 |
| Dydrogesterone ^1^ | 152-62-5 | 312.4 |  | Fipronil sulfone ^1^ | 120068-36-2 | 453.1 |
| Ethisterone ^1^ | 434-03-7 | 312.4 |  | Flufenoxuron ^1^ | 101463-69-8 | 488.8 |
| Ethynodiol diacetate ^1^ | 297-76-7 | 384.5 |  | Fluvalinate ^6^ | 69409-94-5 | 502.9 |
| Etonogestrel ^1^ | 54048-10-1 | 324.5 |  | Heptachlor ^1^ | 76-44-8 | 373.3 |
| Gestodene ^1^ | 60282-87-3 | 310.4 |  | Heptachlor endo-epoxide ^1^ | 28044-83-9 | 389.3 |
| Gestonorone ^4^ | 2137-18-0 | 316.4 |  | Heptachlor exo-epoxide ^1^ | 1024-57-3 | 389.3 |
| Levonorgestrel ^1^ | 797-63-7 | 312.4 |  | Hexachlorobenzene ^1^ | 118-74-1 | 284.8 |
| Lynestrenol ^1^ | 52-76-6 | 284.4 |  | HPTE ^1^ | 2971-36-0 | 317.6 |
| Medroxyprogesterone ^1^ | 520-85-4 | 344.5 |  | Imazalil ^1^ | 35554-44-0 | 297.2 |
| Megestrol acetate ^1^ | 3562-63-8 | 342.5 |  | Lindane ^1^ | 58-89-9 | 290.8 |
| Mifepristone ^1^ | 84371-65-3 | 429.6 |  | Linuron ^1^ | 330-55-2 | 249.1 |
| Nestorone ^1^ | 7759-35-5 | 370.5 |  | Mecoprop ^1^ | 93-65-2 | 214.6 |
| Nomegestrol acetate ^1^ | 58652-20-3 | 370.5 |  | Metalaxyl ^1^ | 57837-19-1 | 279.3 |
| Norethisterone ^1^ | 68-22-4 | 298.4 |  | Metamitron ^1^ | 41394-05-2 | 202.2 |
| Progesterone ^1^ | 57-83-0 | 314.5 |  | Methoxychlor ^1^ | [72-43-5](http://www.commonchemistry.org/ChemicalDetail.aspx?ref=72-43-5) | 345.7 |
| Promegestone ^5^ | 34184-77-5 | 326.5 |  | Metolachlor ^1^ | 51218-45-2 | 283.8 |
| Tibolone ^1^ | 5630-53-5 | 312.4 |  | Mirex ^1^ | 2385-85-5 | 545.5 |
| **Pesticides** |  |  |  | Nicosulfuron ^1^ | 111991-09-4 | 410.4 |
| 2,4'-DDE ^6^ | 3424-82-6 | 318.0 |  | Oxadiazon ^1^ | 19666-30-9 | 345.2 |
| 4,4'-DDE ^1^ | 72-55-9 | 318.0 |  | Oxychlordane ^1^ | 27304-13-8 | 423.7 |
| Alachlore ^1^ | 15972-60-8 | 269.8 |  | Oxyfluorfen ^1^ | 42874-03-3 | 361.7 |
| **Pesticides** |  |  |  | **Pesticides** |  |  |
| Aldicarb ^1^ | 116-06-3 | 190.3 |  | Penconazole ^1^ | 66246-88-6 | 284.2 |
| Aldrin ^1^ | 309-00-2 | 364.9 |  | Pencycuron ^1^ | 66063-05-6 | 328.8 |
| Azimsulfuron ^1^ | 120162-55-2 | 424.4 |  | Pendimethalin ^1^ | 40487-42-1 | 281.3 |
| Bifenox ^1^ | 42576-02-3 | 342.1 |  | Pirimiphos-methyl ^1^ | 29232-93-7 | 305.3 |
| Boscalid ^1^ | 188425-85-6 | 343.2 |  | Pretilachlor ^1^ | 51218-49-6 | 311.8 |
| Bupirimate ^1^ | 41483-43-6 | 316.4 |  | Prochloraz ^1^ | 67747-09-5 | 376.7 |
| Captan ^1^ | 133-06-2 | 300.6 |  | Propiconazole ^1^ | 60207-90-1 | 342.2 |
| Chlordecone ^1^ | 143-50-0 | 490.6 |  | Propyzamide ^1^ | 23950-58-5 | 256.1 |
| Chlorosulfuron ^1^ | 64902-72-3 | 357.8 |  | Tebuconazole ^1^ | 107534-96-3 | 307.8 |
| Chlorpropham ^1^ | 101-21-3 | 213.7 |  | Tefluthrin ^1^ | [79538-32-2](https://www.ncbi.nlm.nih.gov/pcsubstance/?term=%2279538-32-2%22%5bCompleteSynonym%5d%20AND%205281874%5bStandardizedCID%5d) | 418.7 |
| Chlorpyriphos ^1^ | 2921-88-2 | 350.6 |  | Terbutylazine ^1^ | 5915-41-3 | 229.7 |
| Chlortoluron ^1^ | 15545-48-9 | 212.7 |  | Thiabendazole ^1^ | 148-79-8 | 201.3 |
| Cis-chlordane ^1^ | 5103-71-9 | 409.8 |  | Thiacloprid ^1^ | 111988-49-9 | 252.7 |
| Cis-nonachlor ^6^ | 5103-73-1 | 444.2 |  | Thiophanate-methyl ^1^ | 23564-05-8 | 342.4 |
| λ-Cyhalothrin ^1^ | 91465-08-6 | 449.9 |  | Tolclofos-methyl ^1^ | 57018-04-9 | 301.1 |
| Cypermethrin ^1^ | 52315-07-8 | 416.3 |  | Toxaphene ^6^ | 8001-35-2 | 413.8 |
| Cyproconazole ^1^ | 94361-06-5 | 291.8 |  | Trans-chlordane ^1^ | [5103-74-2](https://www.ncbi.nlm.nih.gov/pcsubstance/?term=%225103-74-2%22%5bCompleteSynonym%5d%20AND%2012303039%5bStandardizedCID%5d) | 409.8 |
| Deltamethrin ^1^ | 52918-63-5 | 505.2 |  | Trans-nonachlor ^1^ | 39765-80-5 | 444.2 |
| Diclofop-methyl ^1^ | 51338-27-3 | 341.2 |  | Triclosan ^1^ | 3380-34-5 | 289.5 |
| Dieldrin ^1^ | 60-57-1 | 380.9 |  | Triflumizole ^1^ | 68694-11-1 | 345.8 |
| Diethofencarb ^1^ | 87130-20-9 | 267.3 |  | Vinclozolin ^1^ | 50471-44-8 | 286.1 |
| Diflubenzuron ^1^ | 35367-38-5 | 310.7 |  | Vinclozolin M2 ^6^ | 83792-61-4 | 260.1 |
| Diuron ^1^ | 330-54-1 | 233.1 |  | Ziram ^1^ | 137-30-4 | 305.8 |
| Endosulfan ^1^ | 115-29-7 | 406.9 |  |  |  |  |

^1^ Sigma Aldrich; ^2^ Tocris biosciences; ^3^ Axon; ^4^ Steraloids; ^5^ Sanofi gift; ^6^ Santa Cruz Biotechnology.

**Supplementary Table 2.** RT-PCR primers containing XhoI and KpnI restriction enzyme sites for the isolation of total zfPXR coding sequence (M1-T430) from ZFL cells extracts.

| ZfPXR(ZFL) xho1 S | GGAACTCGAGATGTCCCGCTTATATG |
| --- | --- |
| ZfPXR(ZFL) Kpn1 AS | GGAAGGTACCTTAGGTGTCTTTGCTTAT |

**Supplementary Table 3.** Reporter cell lines used in the study.

| **Reporter cell line** | **Nuclear receptor** | **Reporter gene** |
| --- | --- | --- |
| HG5LN |  | GAL4RE_5_-βGlobin-Luciferase |
| HG5LN Gal4-hPXR | Gal4(DBD)-hPXR(LBD) | GAL4RE_5_-βGlobin-Luciferase |
| HG5LN Gal4-zfPXR | Gal4(DBD)-zfPXR(LBD) | GAL4RE_5_-βGlobin-Luciferase |
| ZFL-zfPXR | Full length zfPXR | PXRE_6_-TATA-Luciferase |

DBD: DNA-binding domain; LBD: ligand-binding domain; RE: response elements.
